# Supplementary material for: Layer-by-Layer Assembly of Polystyrene/Ag for a Highly Reproducible SERS Substrate and Its Use for the Detection of Food Contaminants
Source: Polymers (Basel). 2021 Sep 25;13(19):3270. doi: 10.3390/polym13193270 (PMC8512144; doi:10.3390/polym13193270)
Supplement: Supplementary file 1 [file polymers-13-03270-s001.zip › polymers-1381713-supplementary.pdf]

polymers

Supplementary Information:

Layer-by-layer Assembly of Polystyrene/Ag for a Highly Reproducible SERS Substrate and Its Use for the Detection of Food Contaminants

Sihan Zhang [1], Zhihua Xu [1], Jiaqi Guo [2], Haiying Wang [3], Yibo Ma [4], Xianming Kong [1,*], Hongtao Fan [1] and Qian Yu [1,*]

[1] School of Petrochemical Engineering, Liaoning Petrochemical University, Fushun 113001, China; h321065125@163.com (S.Z.); kxm.kxm@163.com (Z.X.); httyf_77@163.com (H.F.)
[2] Jiangsu Co-Innovation Center for Efficient Processing and Utilization of Forest Resources and Joint International Research Lab of Lignocellulosic Functional Materials, Nanjing Forestry University, Nanjing 210037, China; jiaqi.guo@njfu.edu.cn
[3] School of Environmental Science, Nanjing Xiaozhuang University, Nanjing 210037, China; wanghaiying@nju.edu.cn
[4] Department of Bioproducts and Biosystems, School of Chemical Engineering, Aalto University, FI-00076 Aalto, Finland; yibo.ma@aalto.fi
* Correspondence: xmkong@lnpu.edu.cn (X.K.); qyu@lnpu.edu.cn (Q.Y.)

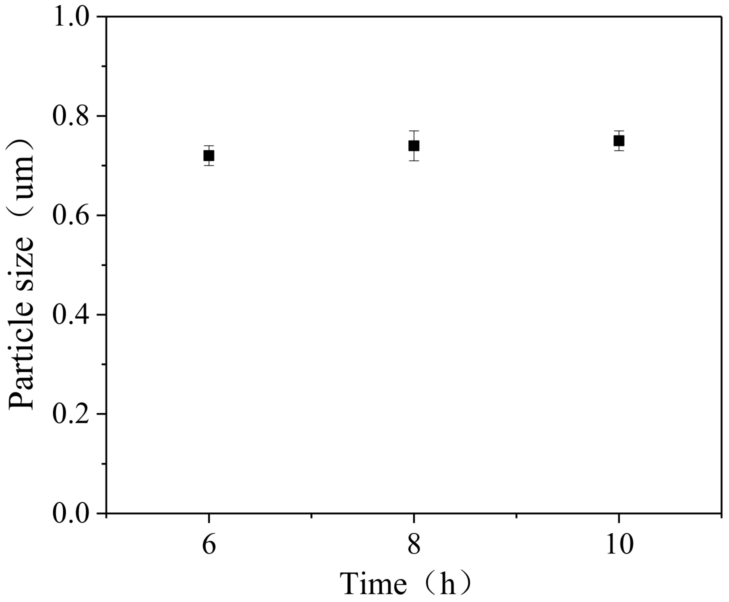

Figure S1. Relation of sphere particle size and reaction time.

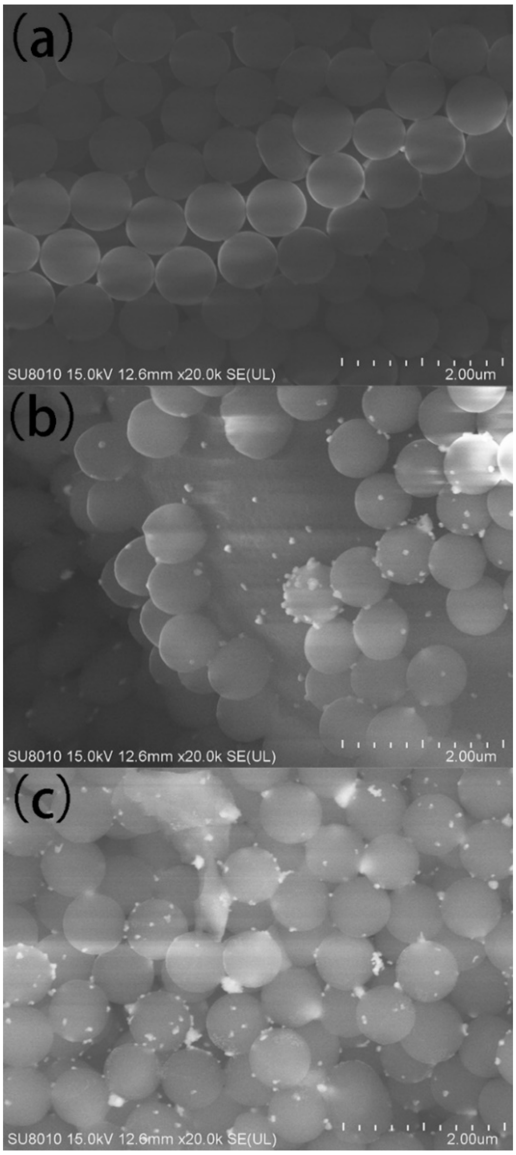

Figure S2. SEM images of colloidal PS/Ag with different volume of PS colloid,400 uL (a), 100 uL (b), 50 uL (c).

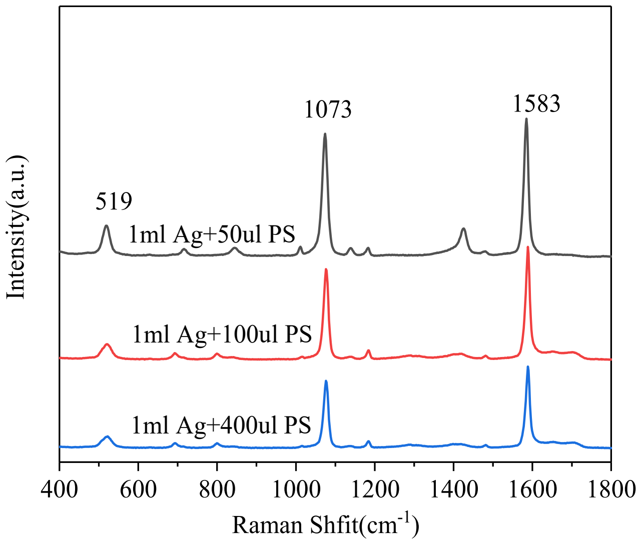

Figure S3. Raman spectra of MBA (100 ppm) on colloidal PS/Ag composites with different volumes of PS colloid.

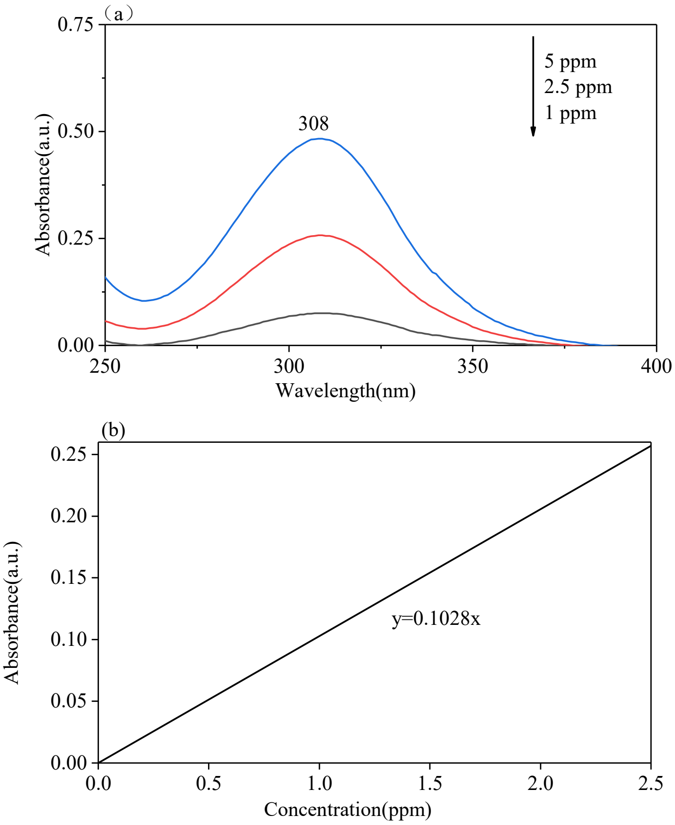

Figure S4. UV-vis spectra of different concentrations of DMZ(a), and the linear relationship between concentration and absorbance (b).

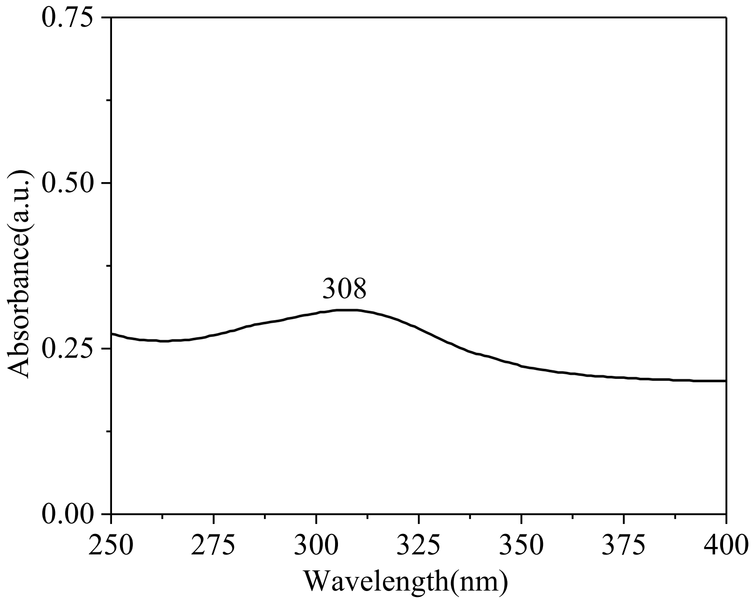

Figure S5. UV-vis spectra of DMZ (100 ppm) from real pork sample after 100-fold dilution.

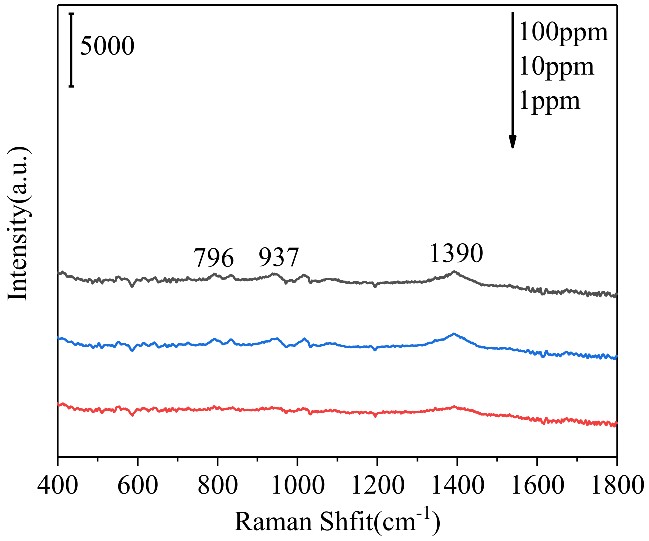

Figure S6. Raman spectra of different concentrations of DMZ from pork after washing with water.
